# Short-term effect of beinaglutide combined with metformin versus metformin alone on weight loss and metabolic profiles in obese patients with polycystic ovary syndrome: a pilot randomized trial

**DOI:** 10.3389/fendo.2023.1156521

**Published:** 2023-06-06

**Authors:** Qing Wen, Song Fang, Yanjing Liang, Yuting Tian, Yiding Chen, Jun Yuan, Qiu Chen

**Affiliations:** ^1^ Medical Department of Endocrinology, Hospital of Chengdu University of Traditional Chinese Medicine, Chengdu, China; ^2^ Medical Department of Endocrinology, The Traditional Chinese Medicine Hospital of Longquanyi, Chengdu, China

**Keywords:** beinaglutide, metformin, polycystic ovary syndrome, obesity, weight loss, gonadal profiles

## Abstract

**Objective:**

To observe the effect of beinaglutide combined with metformin versus metformin alone on weight loss and metabolic profiles in obese patients with polycystic ovary syndrome(PCOS).

**Methods:**

A total of 64 overweight/obese women with PCOS diagnosed *via* the Rotterdam criteria were randomly assigned to metformin(MET) 850 mg twice a day(BID) or combined MET 850 mg BID with beinaglutide (COMB) starting at 0.1mg three times a day(TID)and increasing to 0.2mg TID two weeks later. The main endpoints were changes in anthropometric measurements of obesity. Glucose and lipid metabolic, gonadal profiles, and antral follicle count changes as secondary outcomes were also observed.

**Results:**

60(93.75%) patients completed the study. In terms of lowering weight, body mass index (BMI),waist circumference(WC) and waist to height ratio(WHtR), COMB treatment outperformed MET monotherapy. Subjects in the COMB arm lost weight 4.54±3.16kg compared with a 2.47±3.59kg loss in the MET arm. In the COMB group, BMI,WC and WHtR were reduced significantly compared with that in the MET group, respectively. COMB therapy is also more favorable in the reduction of fasting insulin(FINS), total testosterone(TT), and homeostasis model assessment–insulin resistance(HOMA-IR) when compared to MET therapy. Antral follicle count and ovarian volume were non-significantly changed in both groups.The most frequent side effects in both groups were mild and moderate digestive symptoms. Itching and induration at the injection site were reported with COMB treatment.

**Conclusion:**

Short-term combined treatment with beinaglutide and metformin appears superior to metformin monotherapy in lowering body weight, BMI, WC,WHtR and improving insulin sensitivity and androgen excess in women with PCOS and obesity, with tolerable adverse events.

**Clinical trial registration:**

https://www.chictr.org.cn/listbycreater.aspx, identifier ChiCTR2000033741.

## Introduction

Polycystic ovary syndrome(PCOS) is the most common endocrine disease in women of reproductive age, affecting 6–15% based on the subjects investigated and the diagnostic standards applied (1). The main characteristics of PCOS are hyperandrogenism, oligo-amenorrhea, and polycystic ovarian morphology (2). Obesity is present in 50–80% of women with PCOS, in particular with abdominal obesity, and aggravates the adverse features of the syndrome such as hyperinsulinemia, abnormal glucose tolerance, and a higher vulnerability of type 2 diabetes mellitus(T2DM) and cardiovascular diseases (3). Thus, weight control is the root of treatment in overweight and obese women with PCOS. Even a small weight reduction of 5–10% is quite important for lowering metabolic risk factors and improving reproductive ability (4). Lifestyle intervention is recommended as the first-line therapy for weight management but is difficult for patients to persist. Metformin, an insulin sensitizer, has been proven similar benefits to lifestyle interventions regarding body weight loss and even is superior in improving hyperandrogenism in PCOS women (5). However, weight loss often remains unsatisfactory and non-sustainable with only lifestyle alteration or association with metformin in the clinical practice of PCOS treatment (1). Thus, an alternative strategy is required in the clinical intervention of PCOS for patients who do not respond well to weight loss.

Glucagon-like peptide-1 receptor analogs(GLP-1RAs) administrations on the incretin system are a new therapy for T2DM through improving insulin resistance(IR) and impaired glucose tolerance (6). They have also been proven effective for gradual and long-term weight reduction in other populations with obesity, whether or not they have diabetes (6). Many GLP-1RAs have been created and can be categorized into short-acting and long-acting compounds based on their pharmacokinetic properties. In 2014, the Food and Drug Administration (FDA) authorized long-acting liraglutide for the treatment of obesity (7). Until now, liraglutide and short-acting exenatide have been found to cause weight loss and improved glucose metabolism in the treatment of PCOS (8). Additionally, the published studies provide convincing evidence that liraglutide combined with metformin is substantially more effective than metformin alone in weight loss, improving menstrual cyclicity, ovulation rate, androgens, and insulin sensitivity (9). However, unpleasant gastrointestinal reactions including nausea and vomiting are frequently reported in the use of liraglutide and exenatide which are identical to human GLP-1 in their linear amino acid sequence by 97% and 53%, respectively (10, 11).

Beinaglutide is a short-acting recombinant human GLP-1 (rhGLP-1) with about 100% homology to human GLP-1 (7–36). It is an approved treatment for T2DM according to the Chinese Guideline for the Prevention and Treatment of T2DM (2017 edition) (12). Different homology to human GLP-1 (7–36) among GLP-1RAs may result in various actions, efficacy, and tolerability of these medications in the administration. Clinical studies have shown that beinaglutide was effective in decreasing body weight and improving glycaemic control in overweight and obese T2DM patients (12). Animal research has found that beinaglutide reduces body weight gain caused by high-fat diets, fat storage in adipose tissues, and harm to blood lipid and hormone profiles linked to obesity (7). However, there is very little information on the efficacy and safety of beinaglutide in obese PCOS patients. Moreover, metformin has a relatively unexplored potential to improve the therapeutic index of GLP-1. We hypothesized that metformin may promote reducing the weight of low-dose beinaglutide and bring some benefits in the glucose and lipid metabolism of PCOS-related obesity in comparison to high-dose metformin monotherapy. The aim of this pilot randomized study was to compare the low-dose beinaglutide combined with metformin to high-dose metformin alone on indices of obesity in obese PCOS patients.

## Methods

### Participants

This was a randomized, prospective, open-label,12-week pilot study recruiting 60 PCOS patients with obesity according to ASRM-ESHRE Rotterdam criteria and was performed exclusively at a single center (Hospital of Chengdu University of Traditional Chinese Medicine, PR China)from August 2018 to December 2019 (13). This research followed the tenets of the Declaration of Helsinki and was approved by the Ethics Committee of the Hospital of Chengdu University of Traditional Chinese Medicine(registration number:2017KL-033). The study was registered with ClinicalTrials.gov (ChiCTR2000033741) on the 15th of May 2020.

Eligible subjects refer to overweight and obese women(BMI≥24kg/m^2^), aged 18-40 years who meet the diagnostic criteria of PCOS and promise to continuously utilize barrier contraception while undergoing treatment. The main inclusion requirement also included no therapy that impairs ovarian or insulin sensitivity throughout the first three months of the study. The exclusion criteria for the participants were as follows:1)combined with diabetes mellitus, pituitary tumors, thyroid dysfunction, adrenal tumors, and other endocrinology diseases;2)abnormal liver function and renal insufficiency (alanine transaminase(ALT)levels 2.5 times higher than the upper limit of the normal range, estimated glomerular filtration rate(eGFR)<60mL/min/1.73m^2^);3)clinically serious diseases of the cardiovascular and hematopoietic system, etc;4)menstrual abnormalities caused by congenital deficiencies or organic lesions such as congenital malformations of reproductive organs and gonadal hypoplasia;5)pregnancy or lactation;6)had a previous episode of acute pancreatitis;7)allergy to GLP-1RAs or metformin;8) did not take the medication as prescribed, or could not judge the therapeutic effect, or could not cooperate with the follow-up;9)usage of injectable or oral hormonal contraceptives within 6 months, other steroid hormones, drugs that affect endocrine indicators and insulin sensitivity, and/or anti-obesity medications before 3 months of study enrollment.

Our medical staff addressed 82 subjects to find and enroll potential research participants, and 75 of them voluntarily provided written informed consent. At the initial examination, participants were screened with liver and kidney function biochemical tests, blood lipid profile, fasting blood glucose, 2h postprandial blood glucose, thyroid stimulating hormone, prolactin, testosterone, and qualitative β human chorionic gonadotropin (β-hCG) to exclude increased liver enzymes and impaired kidney function, severe hypertriglyceridemia, diabetes, thyroid disorders, hyperprolactinemia, and/or pregnancy. And they were also questioned about the regularity and length of their menstrual cycle for the last 6 months. Based on their test findings, eleven individuals were determined to be ineligible.64 non-diabetic women who satisfied the requirements for obesity and PCOS were finally recruited.

### Procedures

After an 8–12 hour overnight fast, all laboratory tests were conducted on participants between 6:30 AM and 9:30 AM. All subjects had a standard 75-g oral glucose tolerance test (OGTT) at the start and end of the trial. A baseline blood sample was collected before administering a 75-g oral glucose load. Blood was then drawn 30, 60, and 120 minutes later for assessment of the levels of glucose and insulin. Glucose tolerance can be classified as normal, impaired, or diabetic based on the American Diabetes Association. Hypoglycemia was considered as signs of low blood glucose that were validated by a self-monitored blood glucose measurement of less than 3.9 mmol/L (14). Besides the lipid panel, baseline sex hormone levels (TT, follicle-stimulating hormone(FSH), luteinizing hormone(LH), LH/FSH) were tested using this fasting baseline blood samples uniformly as all of our enrolled patients had irregular menstruation(oligomenorrhea and amenorrhea). Safety clinical assessment was performed at weeks 4, 8, and 12 of the treatment period *via* routine blood, urine, and stool tests; electrocardiography, and liver and kidney function tests. All participants were required to strictly use barrier contraception and keep a bleeding record during the study.

All individuals received standard anthropometric data referring to height, body weight, waist circumference(WC), and waist-to-hip ratio(WHR) taken by a trained physician at baseline and the 12^th^ week after treatment. The weight and height of each patient in light clothing were calculated to the closest 0.01 kg and 0.01 cm, respectively. Body mass index (BMI) was determined as weight (kg) divided by the square of height (m). Hip circumference(HC) was measured at the largest girth and WC measurement was made at the narrowest point between the iliac crest and the lowest rib during minimal respiration (15). The WHR was computed as WC divided by HC in cm. The waist to height ratio (WHtR) was assessed as WC divided by height in cm. The grading of hirsutism was evaluated using a modified Ferriman-Gallwey score (mFG) (16).

Transvaginal three-dimensional ultrasound and transabdominal ultrasound which is only performed in nonsexually active females were carried out to measure the antral follicle count(AFC) and ovarian volume in the early follicular phase or the amenorrhea period at baseline and study completion by a single investigator of the ultrasonic department, Hospital of Chengdu University of Traditional Chinese Medicine. The volume of each ovary was calculated as follows: length*width*height*0.523 (17).AFC and ovarian volume were reported on the larger side of the ovary.

### Randomization

Participants were randomly allocated(1:1) into the COMB arm or MET according to the computer-generated random numbers applying the block randomization method. In the COMB arm, metformin (850mg BID) was given orally to all patients who received metformin, and beinaglutide was given subcutaneously 5 minutes before a meal to all patients who received beinaglutide, starting at 0.1mg TID and increasing to 0.2mg TID two weeks later. After 12 weeks of treatment, the two groups were stopped and followed up for 4 weeks.

### Outcomes

The study’s main endpoints were mean alterations in obesity measurement scales. Changes in hormone levels, antral follicle counts, mFG score, and metabolic levels of glucose and lipids were secondary outcomes. All women were told to report any adverse events *via* direct inquiries, patient self-report, physical exams, and clinical laboratory analysis throughout the treatment. They were advised on recommended lifestyle interventions such as an active promotion of healthy eating and physical activity at the start of the research.

### Assays

A common glucose oxidase technique was used to measure glucose levels (Glucose Reagent Kit, Bayer Newbury, UK). Insulin was determined by solid-phase enzyme-labeled chemiluminescent immunometric assay (Siemens Centaur^®^ XP, Tarrytown, NY, USA). The levels of FSH, LH, TT, and LH/FSH were tested by chemiluminescence immunoassay using Beckman Coulter DXI 800. Lipids were determined using Adiva 1800, Siemens analyzer. An automated kinetic enzyme test was applied to quantify serum creatinine and liver enzymes. The intra-assay and inter-assay coefficients of variations ranged from 1.6%- 6.3% and 5.8% - 9.6% for the applied methods, respectively. Each patient’s pre- and post-intervention serum samples were measured in the same assay run. We applied HOMA-IR=FINS (mIU/L)* fasting plasma glucose (FPG, mmol/L)/22.5 to measure insulin sensitivity (18).

### Statistical analysis

The sample size was calculated by Power and Sample Size Calculation version 3.0.43 (19). In accordance with mean change in weight and data from previous studies with comparable interventions, the sample size of each group required for a statistical power of 80% was 28 patients to determine a significant difference with approximate 2.5 kg in weight loss. 32 individuals were finally enrolled in each group.

We used the Shapiro-Wilk test to assess the data normalization. For continuous data, Mean ± standard deviation(SD) or median(interquartile range)were used to describe normal or non-normal distribution respectively. Nonparametric Wilcoxon signed-rank test(non-normal distribution) and paired-sample t-test(normal distribution) were applied to intra-group values comparisons. To compare the baseline values and the change of clinical variables among inter-groups, the Mann–Whitney test or independent-sample t-test were used for non-normal and normal data, respectively. Classified variables were expressed as percentages (frequencies) and compared by the chi-square test or Fisher exact tests. The level of statistical significance was set at p-values of <0.05 and all p-values were two-tailed. All data analyses were conducted using IBM SPSS Statistics version 24.0.

## Results

### Participants and baseline results

**Figure 1:**
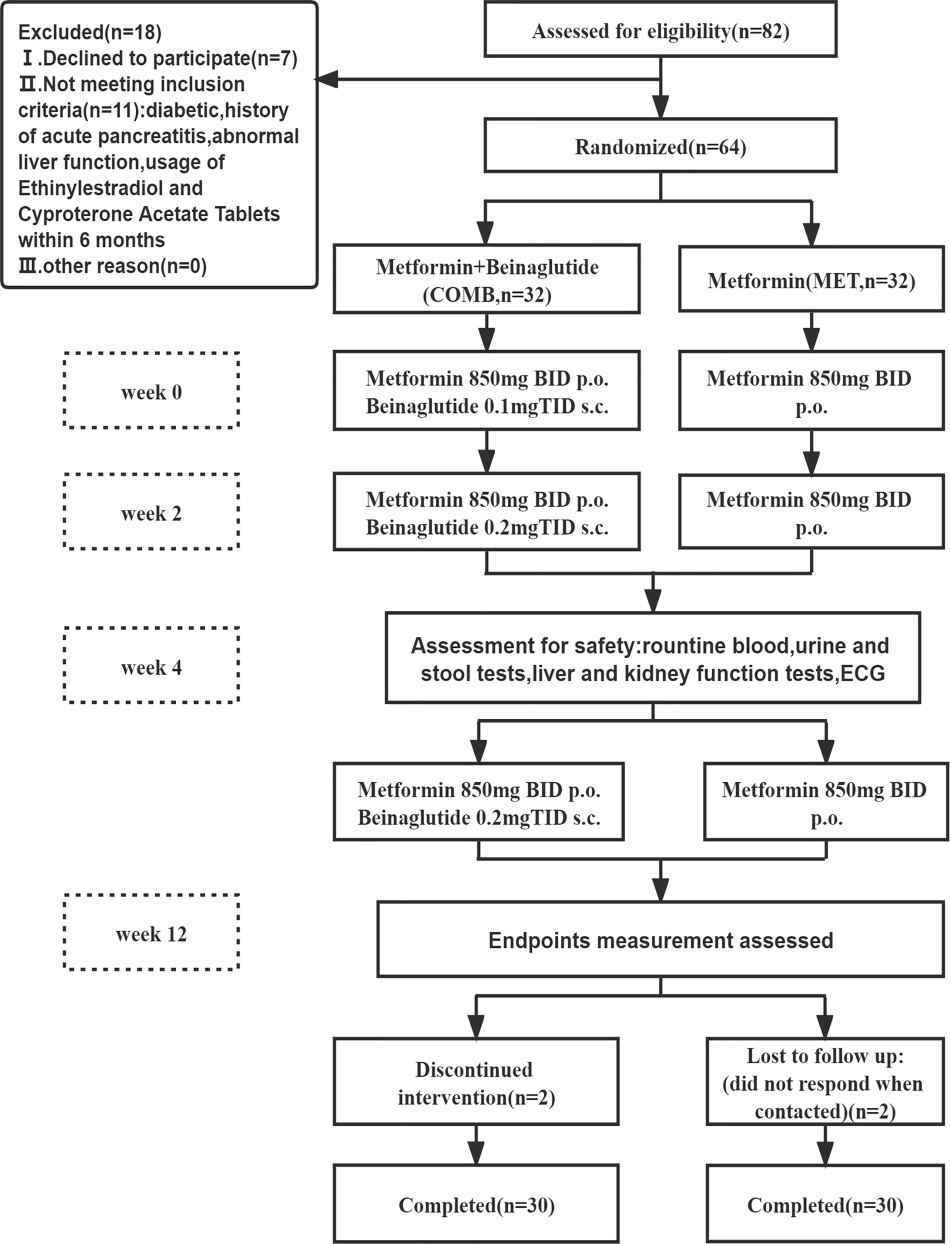
Flow chart of the study design.

Of the 64 overweight/obese women with PCOS starting on treatment,60 patients completed the study (**Figure 1**). Two patients discontinued the study in the COMB group as they were unable to take their medication on time due to business. Two patients missed visits in the MET group during the study period. Baseline characteristics comparisons of all randomized patients were similar in any anthropometric, ovarian ultrasound, hormonal fluctuations, hirsutism score, glycemic, blood lipid levels and Rotterdam phenotype between the two arms (**Table 1**). The pre-treatment and post-treatment parameters of participants completing the trial in each treatment group are described in **Table 2**. **Table 3** presented the comparison changes of the anthropometric and laboratory parameters before and after treatment between the two groups.

**Table 1:** Baseline characteristics of Intent-to-treat participants.

Parameter	COMB(n=32)	MET (n=32)	P
Age (years)	26.75 ± 4.42	25.43 ± 3.14	0.743
Anthropometric			
Weight(kg)	73.95 ± 6.71	72.68 ± 6.23	0.436
BMI (kg/m^2^)	28.65 ± 1.93	28.79 ± 2.12	0.783
WC (cm)	96.45 ± 6.99	89.65(6.85)	0.190
WHR	0.98 ± 0.06	0.96 ± 0.12	0.168
WHtR	0.57 ± 0.05	0.56 ± 0.04	0.060
Hormonal			
TT (nmol/L)	45.28 ± 10.23	44.06 ± 10.11	0.633
FSH(μIU/L)	5.78 ± 1.14	5.83 ± 2.03	0.904
LH(μIU/L)	10.88(6.76)	10.08(4.99)	0.573
LH/FSH	2.73(0.65)	2.35(0.63)	0.265
Hirsutism			
mFG	6.79 ± 2.66	7.05 ± 2.19	0.671
Ovarian ultrasound			
Ovarian volume(mL)	11.82 ± 2.41	10.56 ± 3.35	0.089
AFC	11.45 ± 0.86	11.68 ± 0.82	0.278
Glycemic			
FPG(mmol/L)	5.53 ± 0.35	5.28 ± 0.78	0.105
OGTT 2hPG(mmol/L)	6.28(0.36)	6.09(0.85)	0.542
FINS (μIU/L)	24.38(10.66)	24.12(7.43)	0.655
OGTT 2hINS(μIU/L)	156.25 ± 51.35	152.63 ± 46.21	0.768
HOMA-IR	5.85(2.62)	5.74(2.51)	0.437
Blood Lipid			
TC (mmol/L)	4.76 ± 0.48	4.68 ± 0.25	0.407
HDL-C (mmol/L)	1.07(0.27)	1.12(0.53)	0.283
LDL-C (mmol/L)	3.05 ± 0.25	2.99 ± 0.37	0.452
TG(mmol/L)	2.88 ± 0.83	2.59 ± 0.91	0.188
Rotterdam phenotype%(n)			
HA+OA+PCO (phenotype A)	46.8 (15)	56.2 (18)	0.537
HA+OA(phenotype B)	31.2 (10)	34.3 (11)	
HA+PCO(phenotype C)	15.6 (5)	9.3 (3)	
OA+PCO(phenotype D)	6.2 (2)	0(0)	

Values are presented as Mean±SD, median(interquartile range) and percentages (frequencies); COMB, combination of metformin and beinaglutide; MET, metformin; BMI, body mass index; WC, waist circumference; WHR, waist-to-hip ratio; WHtR, waist to height ratio; TT, total testosterone; FSH, follicle-stimulating hormone; LH, luteinizing hormone; mFG, modified Ferriman–Gallwey score; AFC, antral follicle count; FPG, fasting plasma glucose, OGTT 2hPG, 2-hours plasma glucose after OGTT; FINS, fasting insulin; OGTT 2hINS, 2 hours serum insulin after OGTT; HOMA-IR, homeostasis model assessment–insulin resistance; TC, total cholesterol; HDL-C, high-density lipoprotein cholesterol;LDL-C, low-density lipoprotein cholesterol; TG, triglyceride. HA, Hyperandrogenism; OA, oligo-ovulation or anovulation; PCO, polycystic ovaries.

**Table 2:** Before and after 12-week treatment parameters of the participants with polycystic ovary syndrome in each group.

Parameter	COMB(n=30)			MET(n=30)		
	Pre-treatment	Post-treatment	P	Pre-treatment	Post-treatment	P
Anthropometric						
Weight (kg)	72.97 ± 6.79	68.43 ± 5.92	**<0.001**	72.56 ± 5.93	70.09 ± 3.84	**0.046**
BMI (kg/m^2^)	28.87 ± 2.89	25.95 ± 2.76	**<0.001**	28.99 ± 2.32	27.02 ± 1.01	**0.002**
WC(cm)	96.85 ± 6.92	94.67 ± 5.43	**<0.001**	89.70(7.30)	89.90(7.80)	0.057
WHR	0.99 ± 0.06	0.98 ± 0.06	0.521	0.95 ± 0.13	0.94 ± 0.12	0.586
WHtR	0.58 ± 0.04	0.57 ± 0.03	**<0.001**	0.57 ± 0.04	0.56 ± 0.04	0.056
Hormonal						
TT(nmol/L)	44.26 ± 10.23	40.49 ± 9.33	**<0.001**	44.25 ± 10.08	42.88 ± 6.75	0.051
FSH(μIU/L)	5.76 ± 0.99	5.84 ± 0.95	0.056	5.79 ± 1.13	5.92 ± 0.81	0.639
LH(μIU/L)	10.82(6.28)	10.11(5.30)	0.262	10.08(4.12)	10.40(3.44)	0.234
LH/FSH	2.83(0.69)	2.51(0.72)	**0.013**	2.75(0.68)	2.64(0.52)	0.155
Hirsutism						
mFG	6.76 ± 2.64	5.49 ± 1.35	0.387	7.08 ± 2.02	6.15 ± 2.11	0.649
Ovarian ultrasound						
Ovarian volume(mL)	11.99 ± 1.23	12.03 ± 1.18	0.834	10.51 ± 2.25	10.37 ± 1.59	0.766
AFC	11.47 ± 0.78	11.37 ± 0.89	0.083	11.63 ± 0.85	11.52 ± 0.47	0.961
Glycemic						
FPG(mmol/L)	5.51 ± 0.33	5.12 ± 0.29	**0.003**	5.29 ± 0.28	5.21 ± 0.84	**0.038**
OGTT 2hPG(mmol/L)	6.51(0.52)	6.86(0.35)	0.152	6.23(1.42)	6.01(0.43)	0.526
FINS (μIU/L)	24.75(10.64)	20.37(8.73)	**0.024**	25.25(10.63)	21.56(8.11)	**0.033**
OGTT 2hINS(μIU/L)	156.79 ± 51.64	153.23 ± 51.04	0.597	152.77 ± 48.25	153.16 ± 40.88	0.068
HOMA-IR	5.68(2.62)	4.98(2.34)	**<0.001**	5.39(2.52)	4.96(3.71)	0.054
Blood Lipid						
TC (mmol/L)	4.81 ± 0.45	4.77 ± 0.48	0.118	4.52 ± 0.63	4.45 ± 0.83	0.159
HDL-C (mmol/L)	1.07(0.26)	1.11(0.33)	0.164	1.13(0.59)	1.32(0.44)	**0.005**
LDL-C (mmol/L)	3.04 ± 0.38	2.98 ± 0.53	0.136	2.96 ± 0.36	2.91 ± 0.29	0.349
TG(mmol/L)	2.89 ± 0.82	2.68 ± 0.74	**0.036**	2.60 ± 0.75	2.43 ± 0.55	**0.041**

Values are presented as Mean±SD and median(interquartile range). COMB, combination of metformin and beinaglutide; MET, metformin; BMI, body mass index; WC, waist circumference; WHR, waist-to-hip ratio; WHtR, waist to height ratio; TT, total testosterone; FSH, follicle-stimulating hormone; LH, luteinizing hormone; mFG, modified Ferriman–Gallwey score; AFC, antral follicle count; FPG, fasting plasma glucose, OGTT 2hPG, 2-hours plasma glucose after OGTT; FINS, fasting insulin; OGTT 2hINS, 2 hours serum insulin after OGTT; HOMA-IR, homeostasis model assessment–insulin resistance; TC, total cholesterol; HDL-C, high-density lipoprotein cholesterol; LDL-C, low-density lipoprotein cholesterol; TG, triglyceride. Bolded P values indicate less than 0.05,statistically significant.

**Table 3:** Changes of parameters of the participants with polycystic ovary syndrome in each group.

Parameter	COMB(n=30)	MET(n=30)	P
Anthropometric			
Weight (kg)	-4.54 ± 3.16	-2.47 ± 3.59	**0.021**
BMI (kg/m^2^)	-2.92 ± 1.48	-1.97 ± 0.81	**0.003**
WC(cm)	-0.65(3.48)	-0.30(1.40)	**0.026**
WHR	-0.01(0.04)	-0.02(0.03)	0.135
WHtR	-0.00(0.02)	-0.00(0.01)	**0.031**
Hormonal			
TT (nmol/L)	-3.38 ± 4.16	-0.10 ± 0.28	**0.003**
FSH(μIU/L)	0.08 ± 0.25	0.13 ± 0.39	0.557
LH(μIU/L)	-0.11(0.25)	-0.14(0.27)	0.148
LH/FSH	-0.14(0.28)	-0.08(0.25)	0.239
Hirsutism			
mFG	-1.27 ± 1.59	-0.93 ± 1.87	0.451
Ovarian ultrasound			
Ovarian volume(mL)	0.04 ± 1.28	-0.15 ± 2.03	0.666
AFC	-0.12 ± 0.67	-0.11 ± 0.94	0.962
Glycemic			
FPG(mmol/L)	-0.39 ± 0.25	-0.06 ± 0.72	0.721
OGTT 2hPG(mmol/L)	-0.09(0.41)	-0.12(0.45)	0.065
FINS (μIU/L)	-2.02(4.13)	-1.86(5.29)	**0.038**
OGTT 2hINS(μIU/L)	-3.56 ± 48.73	0.39 ± 46.24	0.749
HOMA-IR	-0.94(0.62)	-0.27(0.86)	**0.025**
Blood Lipid			
TC (mmol/L)	-0.04 ± 0.58	-0.07 ± 0.69	0.586
HDL-C (mmol/L)	0.04(0.12)	0.13(0.26)	0.069
LDL-C (mmol/L)	-0.06 ± 0.21	-0.05 ± 0.25	0.538
TG(mmol/L)	-0.21 ± 0.61	-0.18 ± 0.63	0.674

Values are presented as Mean ± SD and median(interquartile range). COMB, combination of metformin and beinaglutide; MET, metformin; BMI, body mass index;WC, waist circumference; WHR, waist-to-hip ratio; WHtR, waist to height ratio;TT, total testosterone; FSH, follicle-stimulating hormone; LH, luteinizing hormone; mFG, modified Ferriman–Gallwey score; AFC, antral follicle count; FPG, fasting plasma glucose, OGTT 2hPG, 2-hours plasma glucose after OGTT; FINS, fasting insulin; OGTT 2hINS, 2 hours serum insulin after OGTT; HOMA-IR, homeostasis model assessment–insulin resistance; TC, total cholesterol; HDL-C, high-density lipoprotein cholesterol; LDL-C, low-density lipoprotein cholesterol; TG, triglyceride. Bolded P values indicate less than 0.05,statistically significant.

### Measures of obesity

Body weight and BMI were significantly decreased in both COMB and MET groups (all p-values<0.01). Participants in the COMB group reduced on average 4.54 ± 3.16 kg compared with a 2.47 ± 3.59 kg weight reduction in the MET group (p=0.021, **Table 3**). In the COMB arm, BMI decreased by 2.92 ± 1.48 kg/m^2^ compared to 1.97 ± 0.81 kg/m^2^ in the MET arm with statistically significant treatment differences (p=0.003). WC and WHtR were just decreased in the COMB arm(p<0.001, **Table 2**). There were no obvious changes in WHR in both groups after the 12-week intervention (**Table 2**). The COMB treatment was significantly more effective in improving obesity than only metformin treatment with lower body weight, BMI,WC and WHtR(p<0.05, **Table 3**).

### Hormonal changes and hirsutism score

TT was substantially lower after COMB treatment than after MET (**Table 3**; p=0.003). The level of TT decreased significantly from 44.26 ± 10.23nmol/L to 40.49 ± 9.33nmol/L compared with the metformin group wherein the TT slightly decreased from 44.25 ± 10.08 nmol/L to 42.88 ± 6.75nmol/L as illustrated in **Table 2**. The LH/FSH level was significantly improved by COMB therapy but not by MET treatment(p=0.013, **Table 2**). Whereas there were no significant differences in the changes within-treatment between the two arms (**Table 3**). The FSH, LH level, mFG score, ovarian volume, and AFC were not significantly changed by the treatments (**Table 2**).

### Glucose metabolism measures

Glycemic parameters significant decreases of glucose at 0 min(fasting plasma glucose, FPG) and insulin at 0 min(fasting insulin, FINS) of OGTT were observed by both treatments (p= 0.003, p= 0.038, p= 0.024 and p= 0.033 respectively, **Table 2**), whereas COMB was superior in the decrease of FINS level (p=0.038, Table3). HOMA-IR, which reflects fasting insulin sensitivity, was significantly improved by COMB treatment while not altered by metformin therapy (**Table 2**). In comparison to the MET group, The values of HOMA-IR were decreased to a statistically higher degree with COMB treatment (p=0.025, **Table 3**). The levels of glucose and insulin at 120min of OGTT(OGTT 2hPG, OGTT 2hINS) were not differentially affected by both treatments (**Table 2**). And the between-treatment difference of these two parameters was not statistically significant (**Table 3**).

### Blood lipid measures

Triglyceride(TG) levels decreased significantly in both groups(p=0.036 and p=0.041, **Table 2**), but no distinct between-treatment differences were found(p=0.674, **Table 3**). Total cholesterol(TC) and low-density lipoprotein cholesterol(LDL-C) levels were not consistently changed with any treatment (**Table 2**). High-density lipoprotein cholesterol(HDL-C) level increased significantly with metformin monotherapy(p=0.005, **Table 2**). In short, The COMB therapy did not show higher efficacy than only metformin in regulating blood lipids (**Table 3**).

### Adverse events

The most frequently reported side effects in the MET group were gastrointestinal reactions such as diarrhea(8/32), nausea(13/32), vomiting (2/32), and abdominal distension (10/32) which usually occurred in the first 4 weeks of therapy. Most side effects were gradually resolved from the 8th to12th week. In the COMB group, subcutaneous induration with a diameter of less than 1.0cm at the injection site was most commonly documented with an incidence of 46%(15/32) in the first month after the start of beinaglutide injection, accompanied with intermittent itching. 13 patients reported the subcutaneous nodules faded to complete disappearance after about one month, while 2 patients still had about 2 mm of hyperpigmentation on the abdomen at the end of the follow-up period. The second most common was pruritus locally at the injection site with an incidence of 40%(13/32). In the first 4 weeks of COMB treatment,10/32 individuals had slight digestive problems (nausea and vomiting). However, there was no statistical difference in the incidence of gastrointestinal side effects between the two groups(both P>0.05, **Table 4**). 4/32 women reported other adverse events. Mild headaches were reported by three, mild fatigue by one. Hypoglycemia event was not reported in any group. Some patients in both treatment groups had multiple adverse events. Adverse events were not specified by 9/32 in the MET arm and 7/32 in the COMB arm. The injection method of beinaglutide did not significantly increase withdrawal or decrease adherence over the MET group due to topical skin reactions during the study.

**Table 4:** Adverse events inclusive of ITT.

Items	COMB(n=32)	MET(n=32)	P
Diarrhea	–	8/32(25)	- -
Vomiting	7/32(21)	2/32(6)	0.150
Nausea	8/32(25)	13/32(40)	0.183
Abdominal distension	–	10/32(31)	–
headaches	3/32(9)	–	–
fatigue	1/32(3)	–	–
Injection site pruritus	13/32(40)	–	–
Subcutaneous induration	15/32(46)	–	–

Data are presented as n/N (%). COMB, combination of metformin and beinaglutide; MET, Metformin. ITT, intention-to-treat analysis.

## Discussion

The recent introduction of several medicines for the treatment of diabetes mellitus has increased the therapeutic choices of PCOS. Consideration of GLP-1RAs as the potential therapeutic modality of PCOS has grown (8, 20). This study clearly demonstrated that treatment of overweight/obese PCOS women with beinaglutide 0.2 mg TID and metformin 850 mg BID compared with metformin monotherapy for 12 weeks promotes weight reduction, improved hyperandrogenism, and better glycemic metabolism.

Beinaglutide is a recombinant human GLP-1 with the same human-derived amino acid sequence (7). Zhang et al. thought beinaglutide may exert its beneficial role in mice possibly through stimulating GLP-1R-dependent 3′5′-cyclic adenosine monophosphate (cAMP) formation in the HEK 293 cell, leading to enhancement of glucose-stimulated insulin release in pancreas islets (7). Moreover, the Tmax of beinaglutide was proved about 5min, which is near to the half-life of the endogenous GLP-1 about 1.5-5min (21). Consistent with other GLP-1RAs such as liraglutide and semaglutide (22, 23), beinaglutide also reduces body weight by prolonging gastric emptying period and reducing food intake. GLP-1 receptors are known to be widely distributed in the central nervous system, including the hypothalamic nuclei, hindbrain nuclei, hippocampus and several nuclei in the spinal cord. LP-1RAs could bind to central receptors to inhibit the brain’s feeding center and reduce food intake (24, 25). It seems that weight loss could be beneficial to clinical(reproductive and metabolic) outcomes in PCOS patients (26, 27). Although many studies have confirmed the weight reduction effects of metformin and GLP-1RA (15, 28, 29), the potential action of beinaglutide combined with metformin versus metformin alone on weight loss in PCOS has not yet been studied.

In this present study, the combination therapy of metformin and beinaglutide demonstrated synergistic larger effects on the loss of body weight and BMI. Weight loss ranging from 1.38-7.7kg is consistent with previous studies involving obese patients using GLP-1RAs (6, 30, 31). Zhang et al. found that beinaglutide therapy 3 months had a significant weight loss of about 10.05kg from baseline for 314 diabetic patients in a real-world setting (12). Similarly, Sever et al. observed that the combination therapy of MET 1000mg BID and liraglutide 1.2mg QD compared with MET 1000mg BID monotherapy was more effective in promoting the loss of weight and BMI in obese women with PCOS (6). The average weight loss was 1.2kg in the MET group and 6.5kg in the combination group (6). The difference in weight change is largely ascribed to the various subjects’ initial weight and drug half-lives of GLP-1RAs.At least, we could believe the combined treatment was superior to metformin monotherapies in weight loss, which is probably associated with the additive effect of metformin to beinaglutide by modulation of incretin axis and promotion of the expression of GLP-1 receptor and connected insulinotropic receptors (6, 32, 33).

There is no standard treatment for PCOS which is only addressed symptomatically currently (34). Given the major impact that abdominal adiposity exerts on adverse metabolic comorbidities and infertility risk of PCOS (35), WHR and WC which are well-known to be reliable indicators of abdominal adiposity were measured in this study. Current literature indicates that metformin and GLP-1RAs are often used in the treatment of PCOS due to the improvement of fat distribution and reducing abdominal circumference (36, 37). In a study by Sever et al. (6), 12-week combined treatment with liraglutide(1.2 mg QD) and metformin(1000 mg BID) was associated with a significant decrease in WC in obese women with PCOS on metformin monotherapy. WC reduction with COMB therapy was 5.5 ± 3.8 cm compared with 3.2 ± 2.9 cm with liraglutide monotherapy and 1.6 ± 2.9 cm with MET monotherapy (6). Furthermore, 12-week combined treatment with exenatide and metformin outperformed metformin alone in reducing WC(COMB therapy 4.63 ± 4.42cm vs.MET alone 1.72 ± 3.07cm, p=0.023) (28). These results are perfectly consistent with the finding of our study. Beinaglutide-metformin combined treated subjects WC reduce -0.65(3.48)cm vs.-0.30(1.40)cm with metformin monotherapy.

The pathology mechanisms of PCOS is a vicious circle of hyperandrogenism facilitating abdominal visceral adiposity which aggravates androgen secretion of ovarian and/or adrenal origin by the direct modulation of cytokines such as tumor necrosis factor, interleukin 6, and leptin or the indirect induction of insulin resistance and hyperinsulinism (34). Androgen excess secretion is a prerequisite for PCOS development which arises from dysregulation of the hypothalamus-pituitary-ovary axis(HPOA), leading to sensitize ovarian steroidogenesis in response to enhancement of LH and P450c 17α-hydroxylase hyperactivity of follicular theca cells. Subsequently, elevated levels of androgens account for the maturation arrest of FSH-sensitive follicles and can not grow to normal size, causing ovulatory dysfunction (29). The observation that an imbalance ratio of LH/FSH is one of the common hormonal changes of HPOA function disorders and LH/FSH>2 may have enhanced the activity of adrenal-derived androgens, worsening hyperandrogenemia symptoms such as hirsutism and acne in PCOS patients (4, 29). Hence, lowering high androgen levels and recovering HPOA normal rhythm are necessary to improve hyperandrogenism symptoms and ovarian dysfunction in obese patients with PCOS. The researchers found the advantages of liraglutide and metformin combination therapy over metformin alone therapy for enhancing spontaneous pregnancies and *in vitro* fertilization pregnancy rates, though body weight and fatty tissue decreased similarly with both treatment groups (38). In addition to adipose tissue and bioavailability of androgens reduction, GLP-1RAs have contributed to the improvement of fertility by regulating LH to normal levels and correcting LH abnormalities caused by HPOA axis disturbances and hyperinsulinemia (2).

The improvement of hormonal disorder was most dramatic with COMB therapy wherein the TT and LH/FSH levels decreased over 12 weeks. However, hormonal disturbances were not consistently improved on metformin treatment in our study. Although COMB treatment was associated with a marked reduction in LH/FSH ratio in our study, no significant between-treatment difference exists in both groups. In accordance with our findings, Jensterle et al. found improved TT with the liraglutide. They treated 43 overweight women with oligo-ovulatory PCOS with liraglutide, or combined with metformin for 12 weeks and found the combined treatment markedly reduce TT level, while no change was found with liraglutide monotherapy (39). Another research by Elkind-Hirsch et al. found that after 24 weeks of therapy with the combination of exenatide and MET, the TT levels also considerably reduced (9). This result differed from that of Chuan Xing et al (29)and Liu et al (40), who randomized overweight/obese PCOS patients to receive exenatide or liraglutide combined with MET versus MET alone for 12 weeks and found that neither group saw a clear decrease in the TT levels. There is now widespread agreement that the most sensitive indicator of hyperandrogenaemia is blood concentrations of free testosterone, which exerts biological effects on tissues not by TT (41), And sex hormone-binding globulin (SHBG) as a marker of IR and metabolic complications in PCOS is widely advocated (15). It is well known that the calculation of free testosterone concentrations could be achieved by circulating concentrations of SHBG and TT (34). We are unable to draw overwhelming conclusions regarding these hormonal alterations as a result of the methodological problems with measuring androgens in PCOS, the limited sample size, and the absence of any direct evidence on the effects of beinaglutide on SHBG secretion.

Given that polycystic ovarian morphology was included as a diagnostic criterion from the Rotterdam Consensus Conference in 2003 (42), antral follicle counts and ovarian volume per ovary were evaluated by ultrasound in our study. We did not observe any beneficial effect on ovarian morphology. While participants in the liraglutide group had significant reductions in ovarian volume(-2.0ml) and stroma volume(-1.9ml) compared to no alterations in these parameters in the placebo group in a 26-week randomized clinical trial of obese PCOS women (43). Graafian follicles and progeny were observed to be enhanced in an animal investigation where native GLP-1 administration during the proestrus phase promoted the amplitude of the LH surge and progesterone in the luteal phase (44). However, the ovulatory function should be assessed comprehensively by multiple manifestations including frequency and regularity of menstrual cycles, ovulation, pregnancy rate, and outcome. Thus, the efficacy of beinaglutide in regulating ovarian function needs further investigation.

IR plays a significant role in the metabolic impairment of PCOS, which comes with hormonal imbalances, worsening each other in a vicious cycle of PCOS pathogenesis (2). Obesity and its characteristics, such as excess visceral fat, chronic inflammation, and reactive oxygen species, are responsible for IR in obese women with PCOS by increasing the impaired insulin receptor-related signaling pathways (2). Metformin improves hepatic insulin sensitivity and FPG through the inhibition of the gluconeogenesis of hepatic glycogen and the enhancement of glucose uptake and utilization by peripheral tissues (45). GLP-1RAs not only enhance insulin secretion signaling cascades by increasing cAMP levels in beta cells but also reduce the oxidative stress level *via* increasing nuclear factor erythroid 2-related factor 2(Nrf2) protein expression and preventing the interaction of advanced glycation end products with their receptors, leading to improved insulin sensitivity (2). Other potential mechanisms on how GLP-1RAs increase insulin sensitivity were reported that the enhancement of expression of glucose transporter type 4(GLUT-4) in insulin-dependent tissues, up-regulation of phosphorylated insulin receptor-beta, insulin receptor substrate-1, Akt/GSK-3β in adipocytes, promotion of Akt phosphorylation and protein expression of cyclins A, D1 and E and reduction of endoplasmic reticulum stress (2, 13).GLP-1RAs also activate GLP-1 receptors on beta cells and mimic endogenous GLP-1 which directly acts on the pancreatic islets and inhibits gastric emptying to decrease glucose entry into circulation, thereby reducing fasting and postprandial blood glucose (46). We found that FPG and FINS significantly decreased in both groups while FINS and HOMA-IR were more significant in the COMB group, which confirms the synergistic effect on β-cell function between metformin and GLP-1 receptor agonists, as Derosa et al. (10)reported that exenatide combined with MET was beneficial in protecting β-cells. Xing et al. observed that FPG, FINS, and HOMA-IR were considerably lowered in both the metformin monotherapy and the combination therapy of liraglutide and metformin groups after 12 weeks treatment of PCOS women but no significant differences between the two groups (29). The varied results of glucose metabolism alteration found in various studies may be owing to the small sample size and short duration. Additionally, large variability in IR estimated by HOMA-IR score in women with PCOS and relatively low sensitivity of the HOMA-IR score method were shown in other trials (11).

Androgen excess has been found to increase the production of free fatty acids and inflammatory cytokines, which are substantially linked to dyslipidemia and impacts 70% of PCOS patients (15). Increased TG and reduced HDL-C were considered relatively common dyslipidemia in PCOS (15). We found that TG concentrations were markedly reduced in both schemes although no significant difference between the groups. The MET monotherapy was associated with the augment of HDL-C 0.13(0.26)mmol/L whereas there was no between-treatment group difference. This observation was supported by a previous study that found that combined therapy with exenatide and metformin caused TG levels to decrease significantly in overweight women with PCOS (9).HDL-C and LDL-C levels also did not change dramatically with the combined therapy (9).In another trial with GLP-1RAs add on to metformin versus metformin alone in obese women with PCOS, the lipid profile of patients remained unchanged (6). This difference was likely due to no lifestyle intervention in that study and different metformin dosages. Current research has observed that GLP-1RAs have an impact on the metabolism of adipose tissue in both aspects, by directly regulating preadipocyte differentiation and by mediating lipid metabolism processes to lower lipogenesis and stimulation of lipolysis (2).Beinaglutide has been shown to affect significantly the composition of several lipid categories, such as glycerolipids, glycerophospholipids, and sphingolipids, as well as expression of genes in lipid metabolic pathways in the fat tissues of diet-induced obese mice (21). However, there is still a lack of other clinical studies of beinaglutide in combination with metformin versus metformin in PCOS women for direct comparison, coupled with the bias of small sample size and short observation period in our study. Therefore, a long-term study is required to further assess the effectiveness of beinaglutide combined with metformin in lipid metabolism.

Treatment with GLP-RAs and metformin frequently results in gastrointestinal adverse effects including nausea, vomiting, and diarrhea. They often occurred in the first 4 weeks of treatment with mild to moderate intensity and subside over time. It is noteworthy that previous studies investigating combination therapy of liraglutide and metformin did not show more frequent cases of nausea or vomiting than studies of liraglutide only (47). In our study, more than 30% of the subjects experienced gastrointestinal symptoms in COMB treatment arms, which are consistent with previous studies of GLP-1RAs with 11-48% incidence of nausea (1). And we believe that future research should be focused on the unaddressed impact of beinaglutide monotherapy compared to COMB treatment on the digestive tract symptom in obese PCOS women. Some studies have found that nausea may increase weight reduction in high doses of liraglutide, but does not a major contributor to the weight reduction seen with liraglutide (1, 48). Given the significant weight loss effect observed in the present study, whether this could be ascribed to these side effects of beinaglutide needs further investigation. Moreover, pruritus and induration at the injection site frequently occurred after subcutaneous injection of beinaglutide TID, but no withdrawal from the study regarding this. These injections do not appear to be a major obstacle.

Our study had some limitations. It was unable to determine the sustainability of weight loss and endocrine metabolic effects by COMB therapy for 12 weeks. The sample size was relatively small in each treatment arm, and larger multi-center randomized studies are required to set a placebo group and a beinaglutide alone group for reducing bias and clarifying the safety profile in obese PCOS women. The lack of information on improvements in free testosterone and SHBG which represent directly the androgen activity of the whole body should be addressed in future studies. Finally, the tolerability of beinaglutide sharing 100% homology with human GLP-1(7–36), versus other GLP-1RAs in the treatment of PCOS patients needs to be further explored.

## Conclusions

This study shows preliminary findings that a 12-week combined treatment with beinaglutide and metformin was more effective in weight reduction and a decrease in BMI and WC compared to metformin alone in obese women with PCOS.COMB treatment was also more effective in the improvement of androgen excess and insulin resistance of PCOS women with obesity, with acceptable short-term side effects. Although this evidence is not definitive, it provides new ideas for combination regimens of PCOS patients who respond poorly to metformin. More large-scale, long-term randomized clinical trials for comparison of beinaglutide TID with metformin, as well as cost-effectiveness analysis are necessary to guide the use of beinaglutide as a therapy of PCOS.

## Data availability statement

The original contributions presented in the study are included in the article material. Further inquiries can be directed to the corresponding authors.

## Ethics statement

The studies involving human participants were reviewed and approved by the Ethics Committee of the Hospital of Chengdu University of Traditional Chinese Medicine reviewed this study protocol and gave its approval and consent (Approval Code 2017KL-033). The patients/participants provided their written informed consent to participate in this study.

## Author contributions

QW: data analyses, figure preparation, and manuscript preparation. SF: recruit subjects. YL: responsible for the design of randomization, project funding, and study initiation. YT and JY: anthropometric evaluation. QW and QC: responsible for the design of randomization. SF: random allocation. YL and YC: responsible for manual data measurement before treatment. QW and SF: responsible for guidance and statistics. All authors contributed to the article and approved the submitted version.
